# The FIFA World Cup as a Tool for Global Health Diplomacy

**DOI:** 10.1002/puh2.106

**Published:** 2023-07-17

**Authors:** Deborah Oluwaseun Shomuyiwa, Oluwatomisin Temidayo Ajayi, Manatee Jitanan, Shuaibu Saidu Musa, Ernesto R. Gregorio, Emery Manirambona, Monu Tamang, Alhaji Umar Sow, Dorji Ledra, Theerapon Phungdee, Dawa Gyeltshen

**Affiliations:** ^1^ Faculty of Pharmacy University of Lagos Lagos Nigeria; ^2^ Global Health Focus Lagos Nigeria; ^3^ Department of Physical Education Faculty of Education Kasetsart University Bangkok Thailand; ^4^ Department of Nursing Science Ahmadu Bello University Zaria Nigeria; ^5^ Global Health Focus Africa Abuja Nigeria; ^6^ Department of Health Promotion and Education College of Public Health University of the Philippines Manila Philippines; ^7^ College of Medicine and Health Sciences University of Rwanda Kigali Rwanda; ^8^ Department of Physiotherapy Central Regional Referral Hospital Gelephu Bhutan; ^9^ College of Medicine and Allied Health Sciences University of Sierra Leone Freetown Sierra Leone; ^10^ Khesar Gyalpo University of Medical Sciences of Bhutan Thimphu Bhutan; ^11^ Department of Medicine Jigme Dorji Wangchuck National Referral Hospital Thimphu Bhutan

**Keywords:** football, FIFA World Cup, global health, health diplomacy, health systems, sports

## Abstract

International football competitions like the Continental Championships, Leagues, and World Cup serve as the focal point of large‐scale tournaments with an international scope. With specialized local representatives serving as diplomatic envoys, they can represent interests, beliefs, and history across boundaries. The advancement of world health can also be reflected in this representation. Football can play a crucial role in accomplishing various health goals by raising audience knowledge and encouraging behavioral and lifestyle changes. The World Cup may provide a platform for increased interest in the potential for global health diplomacy. A systems approach is needed to contextualize international soccer within the reality of global health. The World Cup for Health's diplomacy should negotiate with stakeholders and oversee their interactions to provide initiatives and activities that support the globally shared and aligned viewpoint on health outcomes. This article seeks to provide a long‐lasting framework for effectively using the Fédération Internationale de Football Association World Cup, an enormously popular sporting event, as a diplomatic instrument for promoting global health.

## BACKGROUND

Football has a rich and fascinating history that has evolved globally. The sport has deep roots in various countries, including China, where the ancient sport of Cuju emerged in the 3rd century BC as one of the earliest forms of football [1]. However, modern football as we know it originated in 19th century Britain, transitioning from a violent and unregulated game called “Folk Football” to a more structured and organized activity. Public schools in England embraced football as a winter game and sought to establish standardized rules of play [1]. Football's popularity proliferated in Europe due to industrialization, urbanization, increased literacy rates, and expanded media coverage. These helped boost the popularity and profitability of football and engendered the adoption of professionalism in many European countries [2], making the global game entertaining and attracting significant spectators.

The global expansion of football necessitated establishing a unifying governing body, forming the Fédération Internationale de Football Association (FIFA) in 1904. Initially, FIFA's authority was limited to overseeing international competitions among a few nations, including Belgium, Denmark, France, Germany, the Netherlands, Spain, Sweden, and Switzerland. Over time, more countries joined the organization, and FIFA's global influence grew significantly throughout the latter half of the 20th century [3]. Football's first official representation on a global stage occurred at the 1908 Olympic Games. Today, international football events, such as Continental Championships, Leagues, and the FIFA World Cup, are prominent platforms for mass gatherings with worldwide impact. The FIFA World Cup, established in 1930, has become the preeminent international football competition and is widely regarded as the greatest sporting spectacle of our time [4]. The 2022 Men's FIFA World Cup final drew nearly 89,000 spectators and garnered around 1.5 billion global views, whereas overall global engagement with the tournament was estimated at approximately 5 billion [5].

Football plays a crucial role in promoting diplomacy as it serves as a platform for representing interests, values, and history across borders. Athletes and organizers act as diplomatic envoys, fostering international connections and understanding [6]. Furthermore, football's impact can extend to global health development. The sport has long been studied in terms of players’ long‐term health and the public health challenges associated with mass gatherings [6]. However, football events’ political and diplomatic dimensions, especially the World Cup, have yet to be clearly defined. Therefore, this commentary examines how the World Cup has been utilized as a tool for health promotion and explores the potential for its future integration into global health diplomacy.

## FOOTBALL AS A TOOL FOR EMPOWERING HEALTH PROMOTION

Football is an effective tool for promoting health, addressing various public health topics, such as mental health, nutrition, cancer, cardiovascular diseases, physical activity, and smoking cessation. Interventions based on football have successfully increased knowledge and fostered positive lifestyle changes [7]. With the growing prevalence of chronic diseases associated with sedentary lifestyles, football has become an essential means of preventing and controlling noncommunicable diseases [8]. Recreational football has been found to improve heart health, regulate blood sugar levels, and positively impact bone density [9]. Additionally, walking football, a popular variant tailored for older individuals, has demonstrated a significant reduction of 9% in body fat mass after 12 weeks [10]. This activity option promotes exercise at a lower intensity and improves exercise tolerance among this demographic.

Football has proven to be an effective model for imparting life skills and discouraging risky behaviors, such as substance abuse, in developing regions like Africa [11]. FIFA's Football for Schools program, funded by the FIFA Foundation and implemented by about 50 member associations, has empowered over 700 million children with targeted life skills and competencies through fun football sessions [12]. Various strategies, such as charity matches, fundraising campaigns, targeted health promotion, and collaboration with stakeholders like the World Health Organization (WHO), have been employed to support these efforts. In March 2017, FIFA implemented a Tobacco‐Free Policy for their events, leading to entirely smoke‐free tournaments and promoting a smoke‐free lifestyle. Recognizing the importance of health education and advocacy, FIFA and WHO signed a Memorandum of Understanding (MoU) in 2019, establishing a collaborative framework for global health initiatives [13]. As part of this collaboration, the Healthy 2022 World Cup Project was launched in partnership with Qatar, the hosts of the 2022 World Cup [14]. The project aims to create a lasting framework for integrating health, security, and well‐being into football and other major sporting events. Its primary pillars include health promotion, encompassing physical activity, and tobacco smoking cessation; health security, focusing on managing health risks; and awareness‐raising through advocacy and actions to safeguard physical and mental health and well‐being.

## THE INTERSECTION OF HEALTH DIPLOMACY AND FOOTBALL

Global health systems and intercontinental collaboration in health development have evolved, emphasizing the importance of global health diplomacy and health policy systems. Governments and stakeholders are adopting health diplomacy practices to achieve global health outcomes, especially in international integration. Although global governance, particularly in sports, is significant in politics, sports diplomacy still needs to be explored. FIFA has implemented strict rules to prevent political interference in national football, ensuring fair play and reducing inequalities. According to FIFA, football must adhere to the principles of good governance, with member nations maintaining independence and being free from any form of political interference [15]. Qatar's hosting of the 2022 World Cup exemplifies the intersection of politics and sport, generating controversies and international tensions.

Global health diplomacy encompasses negotiating for health promotion, establishing new governance mechanisms, forming alliances, and contributing to peace and security [16]. It involves strengthening international engagement, commitment, and capacity, with key stakeholders recognizing its importance for universal health. Global health diplomacy combines public health, international affairs, management, law, and economics, focusing on global policies shaping the health environment [16]. It is particularly relevant in crises, scaling health programs, and addressing inequalities. As an inclusive sport, football exemplifies diverse cultural, institutional, political, and systemic aspects. The World Cup represents a global collaboration for shared socioeconomic development.

Representation, communication, and negotiation are essential themes in health diplomacy [16]. As sports bring people and countries together, they provide a platform for countries to find common ground and establish consensus on accepted goals and frameworks in foreign relations. Football has been a centerpiece of international relations, promoting health, peace, security, and economic development. Germany strategically used the 2006 World Cup as a diplomatic tool to build international relations. South Africa's hosting of the 2010 World Cup showcased African culture, stimulated infrastructure development, and drove economic growth [17]. The event also left a health legacy, including knowledge transfer, enhanced cooperation, inter‐sectoral collaboration, increased resources, and greater confidence in future preparedness for mass gatherings [18].

## FUTURE ACTIONS OF THE WORLD CUP ON GLOBAL HEALTH DIPLOMACY

Promoting the convergence of international football and global health requires establishing a collaborative framework that nurtures sports exchange programs and fosters innovation, creating new avenues for diplomacy. As a significant sporting event, the World Cup offers a distinct opportunity to generate global interest and enhance global health security. Although some argue that the impact of football diplomacy is often exaggerated, limited to mere symbolism or propaganda, FIFA's proactive approach in integrating the World Cup with global health initiatives sets a solid groundwork. This endeavor aims to unite all relevant stakeholders, including the private sector, to support broader endeavors in health promotion. As the governing body of this international competition, FIFA should take charge and assume leadership of the international coalition. Diplomatic efforts around the World Cup should prioritize negotiating health initiatives aligning with global health objectives and active engagement with the global football community.

Leveraging the World Cup as a platform for public health diplomacy presents an opportunity to enhance grassroots perspectives on health, including the management of infodemics. A key strategy is empowering youth and involving them in proactive and sustainable community health initiatives, fostering innovation and creativity at the intersection of sports and health. Engaging in global dialogues and workshops enables us to recognize and effectively utilize the role of sports in addressing political, social, and cultural challenges associated with the FIFA World Cup. This approach ensures the provision of essential resources for community development, combats misinformation, promotes accurate health information, creates safer and more inclusive sports environments, and emphasizes the empowerment of women and marginalized populations. Additionally, this collaborative effort reinforces the significance of investment in player safety and strengthens health support mechanisms.

Policy development and analysis are integral to shaping perceptions, priorities, and commitments within health systems. By framing the World Cup as an international platform for global health, we can guide how the tournament is perceived, understood, and integrated into health resilience and sustainability measures. This policy orientation opens the door for systematically integrating intercontinental football institutions into health governance and international relations. Conducting research on sports governance becomes imperative to ensure equity, justice, and fair representation of international institutions and their global political impact. Although football can be utilized to promote international policies and enhance prestige, it is crucial to maintain fairness within the sport.

## CONCLUSION

In response to the growing need to address global health challenges, it is crucial to reassess the role of football in promoting global health. As the most popular sport worldwide, football possesses the unique ability to unite people, institutions, and diverse perspectives within stadiums, thereby fostering international relations. Leveraging the platform of the World Cup, we can foster a sense of shared responsibility and develop diplomatic practices that contribute to achieving global health outcomes. The Qatar 2022 World Cup has demonstrated a strong commitment to prioritizing health representation, providing an opportunity to coordinate global health initiatives within the realm of football. By establishing a collaborative framework that encourages sports exchange programs and fosters innovation, we can fully unlock the potential of the World Cup for global health diplomacy. In this endeavor, FIFA, as the governing body of the World Cup, should assume a leadership role in integrating global health initiatives into the tournament.

## AUTHOR CONTRIBUTIONS

*Conceptualizations; writing—original draft; writing—review and editing*: Deborah Oluwaseun Shomuyiwa. *Writing—original draft; writing—review and editing*: Oluwatomisin Temidayo Ajayi. *Writing—review and editing*: Manatee Jitanan, Shuaibu Saidu Musa, Emery Manirambona, Monu Tamang, Dorji Ledra, Theerapon Phungdee, and Dawa Gyeltshen.

## CONFLICT OF INTEREST STATEMENT

Deborah Oluwaseun Shomuyiwa, Shuaibu Saidu Musa, Dawa Gyeltshen, and Emery Manirambona are members of the Youth Editorial Board of Public Health Challenges and coauthors of this article. To minimize bias, they have been excluded from all editorial decision‐making related to the acceptance of this article for publication.

## FUNDING INFORMATION

There is no funding in the development of this paper.
